# Supplementary material for: Safety and Efficacy of Intravenous and Intrathecal Delivery of AAV9-Mediated ARSA in Minipigs
Source: Int J Mol Sci. 2023 May 24;24(11):9204. doi: 10.3390/ijms24119204 (PMC10253118; doi:10.3390/ijms24119204)
Supplement: Supplementary file 1 [file ijms-24-09204-s001.zip › ijms-2372351-supplementary.pdf]

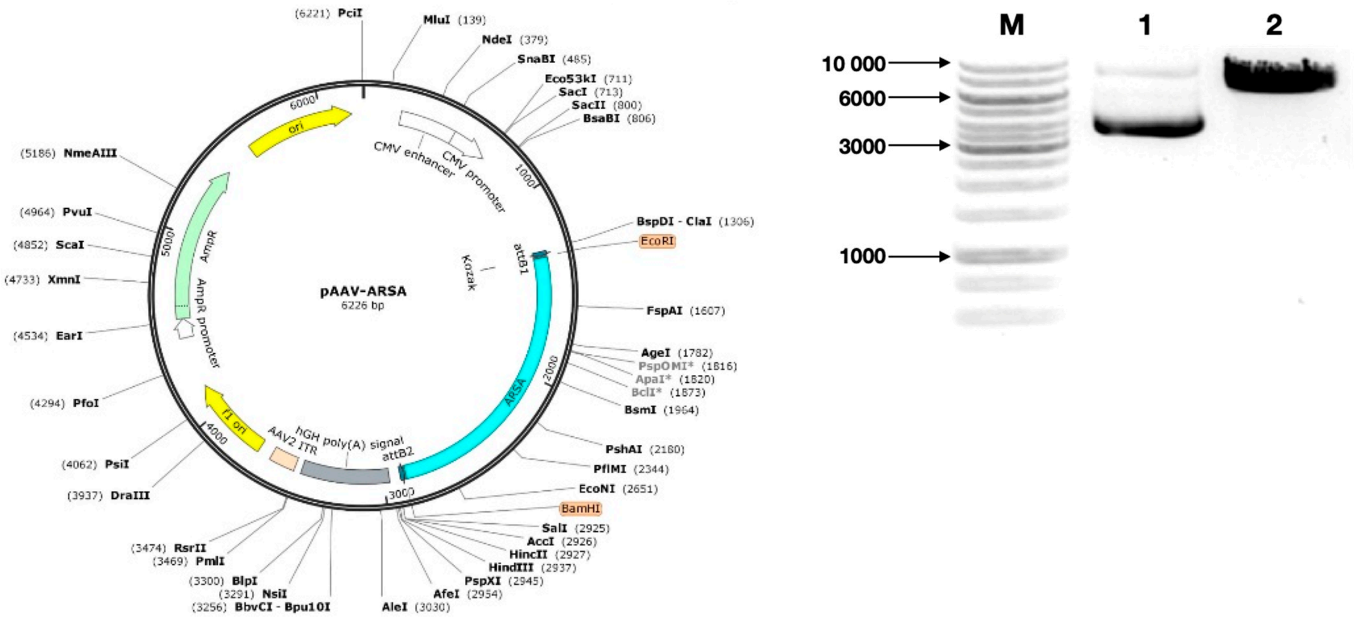

(a) **(b)**

Figure S1. Characterization and analysis of the expression plasmid vector pAAV-ARSA. (**a**) pAAV-ARSA plasmid map; (**b**) Analysis of restriction results for plasmid DNA, M – GeneRullerTM DNA Ladder 1kb marker, 1 – pAAV-ARSA restriction with BamHI enzyme (Cat. No. ER0051, Thermo Fisher Scientific Inc., USA), 2 – pAAV-ARSA cDNA.
